# Supplementary material for: Multidimensional recurrence quantification analysis of human-metronome phasing
Source: PLoS One. 2023 Feb 23;18(2):e0279987. doi: 10.1371/journal.pone.0279987 (PMC9949643; doi:10.1371/journal.pone.0279987)
Supplement: S1 Appendix — (DOCX) [file pone.0279987.s001.docx]

# S1 Appendix: Descriptions of Practice Sessions

**Practice Session 1**

Practice Session 1 familiarized subjects with phasing. Participants practiced phasing with the metronome at 90, 110, and 130 bpm using a visual aid that depicted 𝛹. Participants were instructed to tap in synchrony with the metronome at the beginning of each trial until a warning signal sounded. Then, participants were instructed to increase their tapping rate as slightly as possible, maintain that new tapping rate until completing one phasing lap, synchronize with the metronome again for several beats, and stop tapping. Each trial was programmed to end after 5 seconds of no tapping data. The maximum duration of each trial was 2 minutes. Participants completed a minimum of three trials (i.e., at least one at each tempo) before Practice Session 2.

**Practice Session 2**

Practice Session 2 was identical to Practice Session 1 except that a visual aid was no longer provided. Instead, participants were expected to listen closely to their tapping and the metronome to identify when they had completed one phasing lap. The number of phasing taps and laps were used to compute a score for each trial. Since the goal was to phase gradually and complete only one round of phasing, participants received a score of 100% on trials where they made one lap and met a minimum threshold number of taps (16 taps; experimentally determined from pilot subjects, *N* = 10).

Practice Session 2 included a maximum of 18 trials (i.e., 6 trials at each tempo). The order of stimulus presentation was identical across subjects: 90 bpm for trial one, 110 bpm for trial two, and 130 bpm for trial three. This pattern repeated until participants either scored 100% three consecutive times or completed 18 trials. Participants could move to the experimental trials after scoring 100% on three consecutive trials (i.e., successfully phasing at the range of tempi in the experiment). If participants did not achieve 100% and thus needed to complete all 18 trials of Practice Session 2, they were offered the chance to repeat Practice Session 1 (with visual aid) before continuing to the experiment.
