# Supplementary material for: Multidimensional recurrence quantification analysis of human-metronome phasing
Source: PLoS One. 2023 Feb 23;18(2):e0279987. doi: 10.1371/journal.pone.0279987 (PMC9949643; doi:10.1371/journal.pone.0279987)
Supplement: S2 Appendix — (DOCX) [file pone.0279987.s002.docx]

**Table 5. Linear mixed effect model results for general MdRQA with trial type, music experience, and tempo range as predictors of %REC, %DET, and MAXL.** Each MdRQA metric was predicted using a separate model. Marginal and conditional R^2^ for each model included below each model label. Effect sizes provided as standard estimates (*ß*) for statistically significant predictors.

| **%REC** *Marginal R2 = 0.046, Conditional R2 = 0.106* | | | | | | |
| --- | --- | --- | --- | --- | --- | --- |
| Predictors | Estimate | SE | *df* | *t-value* | *p-value* | *ß* |
| (Intercept) | 42.927 | 0.087 | 53.10 | 495.918 | < 0.0001 | -- |
| Successful trials (v. Incomplete) | 0.035 | 0.198 | 501.85 | 0.176 | 0.860 | -- |
| Unsuccessful trials (v. Incomplete) | 0.137 | 0.201 | 493.56 | 0.680 | 0.497 | -- |
| Musician (v. Nonmusician) | -0.303 | 0.173 | 53.10 | -1.750 | 0.086 | -.14 . |
| Upper tempo range (v. Middle tempo) | 0.098 | 0.125 | 495.97 | 0.782 | 0.434 | -- |
| Lower tempo range (v. Middle tempo) | -0.394 | 0.186 | 487.85 | -2.114 | 0.035 | -.20 * |
| Successful trials x Music exp. | 0.711 | 0.395 | 501.85 | 1.798 | 0.073 | .19 . |
| Unsuccessful trials x Music exp. | 0.825 | 0.402 | 493.56 | 2.051 | 0.041 | .23 * |
| Successful trials x Upper tempo | -0.131 | 0.314 | 496.87 | -0.417 | 0.677 | -- |
| Unsuccessful trials x Upper tempo | 0.045 | 0.335 | 491.00 | 0.135 | 0.893 | -- |
| Successful trials x Lower tempo | 0.198 | 0.540 | 488.45 | 0.366 | 0.715 | -- |
| Unsuccessful trials x Lower tempo | 0.323 | 0.532 | 490.25 | 0.606 | 0.545 | -- |
| Music exp. x Upper tempo | 0.100 | 0.250 | 495.97 | 0.400 | 0.689 | -- |
| Music exp. x Lower tempo | -0.733 | 0.373 | 487.85 | -1.966 | 0.050 | -.19 * |
| Successful x Music exp. x Upper temp. | -0.383 | 0.628 | 496.87 | -0.609 | 0.543 | -- |
| Successful x Music exp. x Lower temp. | 0.933 | 1.080 | 488.45 | 0.864 | 0.388 | -- |
| Unsuccessful x Music exp. x Upper temp. | -0.517 | 0.671 | 491.00 | -0.770 | 0.441 | -- |
| Unsuccessful x Music exp. x Lower temp. | 1.661 | 1.065 | 490.25 | 1.560 | 0.119 | -- |
| **%DET** *Marginal R2 = 0.063, Conditional R2 = 0.083* | | | | | | |
| Predictors | Estimate | SE | *df* | *t-value* | *p-value* | *ß* |
| (Intercept) | 66.126 | 0.204 | 74.94 | 324.351 | < 0.0001 | -- |
| Successful trials (v. Incomplete) | 1.349 | 0.528 | 475.84 | 2.556 | 0.011 | .27 * |
| Unsuccessful trials (v. Incomplete) | 1.077 | 0.535 | 450.58 | 2.015 | 0.044 | .22 * |
| Musician (v. Nonmusician) | -1.132 | 0.408 | 74.94 | -2.775 | 0.007 | -.20 ** |
| Upper tempo range (v. Middle tempo) | 0.084 | 0.339 | 500.89 | 0.249 | 0.803 | -- |
| Lower tempo range (v. Middle tempo) | -1.556 | 0.507 | 491.28 | -3.072 | 0.002 | -.29 ** |
| Successful trials x Music exp. | 4.044 | 1.056 | 475.84 | 3.831 | < 0.0001 | .40 *** |
| Unsuccessful trials x Music exp. | 2.654 | 1.069 | 450.58 | 2.482 | 0.013 | .27 * |
| Successful trials x Upper tempo | 0.183 | 0.851 | 502.35 | 0.215 | 0.830 | -- |
| Unsuccessful trials x Upper tempo | -0.631 | 0.910 | 495.89 | -0.693 | 0.488 | -- |
| Successful trials x Lower tempo | 5.096 | 1.467 | 492.28 | 3.474 | 0.001 | .47 *** |
| Unsuccessful trials x Lower tempo | 4.699 | 1.446 | 494.65 | 3.250 | 0.001 | .45 ** |
| Music exp. x Upper tempo | -0.552 | 0.679 | 500.89 | -0.814 | 0.416 | -- |
| Music exp. x Lower tempo | -2.663 | 1.013 | 491.28 | -2.628 | 0.009 | -.25 ** |
| Successful x Music exp. x Upper temp. | 1.153 | 1.702 | 502.35 | 0.677 | 0.499 | -- |
| Successful x Music exp. x Lower temp. | 12.394 | 2.934 | 492.28 | 4.224 | < 0.0001 | .57 *** |
| Unsuccessful x Music exp. x Upper temp. | -0.094 | 1.821 | 495.89 | -0.052 | 0.959 | -- |
| Unsuccessful x Music exp. x Lower temp. | 9.630 | 2.891 | 494.65 | 3.331 | 0.001 | .47 *** |
| **MAXL** *Marginal R2 = 0.192, Conditional R2 = 0.324* | | | | | | |
| Predictors | Estimate | SE | *df* | *t-value* | *p-value* | *ß* |
| (Intercept) | 8.043 | 0.214 | 35.63 | 37.637 | < 0.0001 | -- |
| Successful trials (v. Incomplete) | -0.832 | 0.366 | 502.88 | -2.271 | 0.024 | -0.23 |
| Unsuccessful trials (v. Incomplete) | -0.507 | 0.374 | 504.89 | -1.356 | 0.176 | -- |
| Musician (v. Nonmusician) | 0.225 | 0.427 | 35.63 | 0.526 | 0.602 | -- |
| Upper tempo range (v. Middle tempo) | 0.367 | 0.229 | 489.62 | 1.605 | 0.109 | -- |
| Lower tempo range (v. Middle tempo) | 0.209 | 0.340 | 485.23 | 0.615 | 0.539 | -- |
| Successful trials x Music exp. | -1.914 | 0.733 | 502.88 | -2.612 | 0.009 | -.27 ** |
| Unsuccessful trials x Music exp. | -2.022 | 0.748 | 504.89 | -2.702 | 0.007 | -.29 ** |
| Successful trials x Upper tempo | 0.761 | 0.574 | 489.86 | 1.324 | 0.186 | -- |
| Unsuccessful trials x Upper tempo | 0.225 | 0.612 | 486.61 | 0.367 | 0.714 | -- |
| Successful trials x Lower tempo | -2.127 | 0.984 | 485.45 | -2.162 | 0.031 | -.27 * |
| Unsuccessful trials x Lower tempo | -2.483 | 0.971 | 486.33 | -2.558 | 0.011 | -.33 * |
| Music exp. x Upper tempo | -0.690 | 0.458 | 489.62 | -1.508 | 0.132 | -- |
| Music exp. x Lower tempo | 1.021 | 0.679 | 485.23 | 1.503 | 0.133 | -- |
| Successful x Music exp. x Upper temp. | -0.268 | 1.149 | 489.86 | -0.233 | 0.816 | -- |
| Successful x Music exp. x Lower temp. | -4.292 | 1.968 | 485.45 | -2.181 | 0.030 | -.27 * |
| Unsuccessful x Music exp. x Upper temp. | -0.526 | 1.223 | 486.61 | -0.430 | 0.667 | -- |
| Unsuccessful x Music exp. x Lower temp. | -5.130 | 1.941 | 486.33 | -2.642 | 0.009 | -.35 ** |

**Table 6. Linear mixed effect model results for region-based MdRQA with trial type, music experience, tempo range, and region as predictors of %REC, %DET, and MAXL.** Each MdRQA metric was predicted using a separate model. Marginal and conditional R^2^ for each model included below each model label. Effect sizes provided as standard estimates (*ß*) for statistically significant predictors.

| **%REC** *Marginal R2 = 0.078, Conditional R2 = 0.085* | | | | | | |
| --- | --- | --- | --- | --- | --- | --- |
| Predictors | Estimate | SE | *df* | *t-value* | *p-value* | *ß* |
| (Intercept) | 20.185 | 0.767 | 23.00 | 26.302 | < 0.0001 | -- |
| Unsuccessful trials (v. Successful) | -7.578 | 1.344 | 818.71 | -5.64 | < 0.0001 | -.20 *** |
| Musician (v. Nonmusician) | -0.128 | 1.535 | 23.00 | -0.083 | 0.935 | -- |
| Region 2 (v. Region 1) | -1.711 | 1.613 | 1178.65 | -1.061 | 0.289 | -- |
| Region 3 (v. Region 1) | -1.447 | 1.613 | 1178.65 | -0.898 | 0.370 | -- |
| Upper tempo range (v. Middle tempo) | -0.910 | 1.648 | 1202.99 | -0.552 | 0.581 | -- |
| Lower tempo range (v. Middle tempo) | -2.859 | 1.420 | 1197.20 | -2.013 | 0.044 | -.07 * |
| Trial type x Music experience | 1.570 | 2.687 | 818.71 | 0.584 | 0.559 | -- |
| Trial type x Upper tempo range | -2.525 | 3.299 | 1201.68 | -0.765 | 0.444 | -- |
| Trial type x Lower tempo range | -6.119 | 2.860 | 1150.95 | -2.139 | 0.033 | -.07 * |
| Music experience x Upper tempo | -1.290 | 3.296 | 1202.99 | -0.391 | 0.696 | -- |
| Music experience x Lower tempo | -2.052 | 2.840 | 1197.20 | -0.723 | 0.470 | -- |
| Trial type x Region 2 | -4.013 | 3.225 | 1178.65 | -1.244 | 0.214 | -- |
| Trial type x Region 3 | 3.223 | 3.225 | 1178.65 | 0.999 | 0.318 | -- |
| Music experience x Region 2 | -0.280 | 3.225 | 1178.65 | -0.087 | 0.931 | -- |
| Music experience x Region 3 | 2.468 | 3.225 | 1178.65 | 0.765 | 0.444 | -- |
| Upper tempo x Region 2 | -2.513 | 4.023 | 1178.65 | -0.625 | 0.532 | -- |
| Lower tempo x Region 2 | -2.012 | 3.472 | 1178.65 | -0.579 | 0.562 | -- |
| Upper tempo x Region 3 | -3.397 | 4.023 | 1178.65 | -0.844 | 0.399 | -- |
| Lower tempo x Region 3 | -1.124 | 3.472 | 1178.65 | -0.324 | 0.746 | -- |
| Trial type x Music exp. x Upper tempo | 1.207 | 6.598 | 1201.68 | 0.183 | 0.855 | -- |
| Trial type x Music exp. x Lower tempo | 1.334 | 5.720 | 1150.95 | 0.233 | 0.816 | -- |
| Trial type x Music exp. x Region 2 | -3.384 | 6.450 | 1178.65 | -0.525 | 0.600 | -- |
| Trial type x Music exp. x Region 3 | 0.864 | 6.450 | 1178.65 | 0.134 | 0.894 | -- |
| Trial type x Upper tempo x Region 2 | 1.440 | 8.046 | 1178.65 | 0.179 | 0.858 | -- |
| Trial type x Lower tempo x Region 2 | 10.954 | 6.944 | 1178.65 | 1.578 | 0.115 | -- |
| Trial type x Upper tempo x Region 3 | 6.989 | 8.046 | 1178.65 | 0.869 | 0.385 | -- |
| Trial type x Lower tempo x Region 3 | 2.967 | 6.944 | 1178.65 | 0.427 | 0.669 | -- |
| Music exp. x Upper tempo x Region 2 | 9.376 | 8.046 | 1178.65 | 1.165 | 0.244 | -- |
| Music exp. x Lower tempo x Region 2 | 12.086 | 6.944 | 1178.65 | 1.741 | 0.082 | -- |
| Music exp. x Upper tempo x Region 3 | -3.393 | 8.046 | 1178.65 | -0.422 | 0.673 | -- |
| Music exp. x Lower tempo x Region 3 | -9.708 | 6.944 | 1178.65 | -1.398 | 0.162 | -- |
| Trial type x Music exp. x Upper tem. x Reg. 2 | 7.112 | 16.093 | 1178.65 | 0.442 | 0.659 | -- |
| Trial type x Music exp. x Lower tem. x Reg. 2 | 7.981 | 13.888 | 1178.65 | 0.575 | 0.566 | -- |
| Trial type x Music exp. x Upper tem. x Reg. 3 | -8.538 | 16.093 | 1178.65 | -0.531 | 0.596 | -- |
| Trial type x Music exp. x Lower tem. x Reg. 3 | -17.915 | 13.888 | 1178.65 | -1.29 | 0.197 | -- |
| **%DET** *Marginal R2 = 0.086, Conditional R2 = 0.160* | | | | | | |
| Predictors | Estimate | SE | *df* | *t-value* | *p-value* | *ß* |
| (Intercept) | 14.141 | 1.918 | 23.41 | 7.373 | < 0.0001 | -- |
| Trial type | -11.429 | 1.847 | 1191.16 | -6.189 | < 0.0001 | -.21 *** |
| Music experience | 0.490 | 3.836 | 23.41 | 0.128 | 0.900 | -- |
| Region 2 | 1.552 | 2.126 | 1179.83 | 0.73 | 0.466 | -- |
| Region 3 | -5.334 | 2.126 | 1179.83 | -2.509 | 0.012 | -.09 * |
| Upper tempo range | -0.314 | 2.188 | 1190.27 | -0.143 | 0.886 | -- |
| Lower tempo range | -3.545 | 1.880 | 1188.03 | -1.886 | 0.060 | -.06 . |
| Trial type x Music experience | -1.904 | 3.694 | 1191.16 | -0.515 | 0.606 | -- |
| Trial type x Upper tempo range | -1.667 | 4.388 | 1193.21 | -0.38 | 0.704 | -- |
| Trial type x Lower tempo range | 2.958 | 3.836 | 1200.53 | 0.771 | 0.441 | -- |
| Music experience x Upper tempo | 0.720 | 4.375 | 1190.27 | 0.165 | 0.869 | -- |
| Music experience x Lower tempo | 1.790 | 3.760 | 1188.03 | 0.476 | 0.634 | -- |
| Trial type x Region 2 | 3.265 | 4.252 | 1179.83 | 0.768 | 0.443 | -- |
| Trial type x Region 3 | 8.064 | 4.252 | 1179.83 | 1.897 | 0.058 | .07 . |
| Music experience x Region 2 | -4.958 | 4.252 | 1179.83 | -1.166 | 0.244 | -- |
| Music experience x Region 3 | 1.372 | 4.252 | 1179.83 | 0.323 | 0.747 | -- |
| Upper tempo x Region 2 | -0.696 | 5.304 | 1179.83 | -0.131 | 0.896 | -- |
| Lower tempo x Region 2 | -5.794 | 4.577 | 1179.83 | -1.266 | 0.206 | -- |
| Upper tempo x Region 3 | 2.996 | 5.304 | 1179.83 | 0.565 | 0.572 | -- |
| Lower tempo x Region 3 | 1.617 | 4.577 | 1179.83 | 0.353 | 0.724 | -- |
| Trial type x Music exp. x Upper tempo | -7.490 | 8.776 | 1193.21 | -0.853 | 0.394 | -- |
| Trial type x Music exp. x Lower tempo | 7.149 | 7.672 | 1200.53 | 0.932 | 0.352 | -- |
| Trial type x Music exp. x Region 2 | 7.618 | 8.504 | 1179.83 | 0.896 | 0.371 | -- |
| Trial type x Music exp. x Region 3 | 0.809 | 8.504 | 1179.83 | 0.095 | 0.924 | -- |
| Trial type x Upper tempo x Region 2 | -4.073 | 10.608 | 1179.83 | -0.384 | 0.701 | -- |
| Trial type x Lower tempo x Region 2 | 0.029 | 9.154 | 1179.83 | 0.003 | 0.998 | -- |
| Trial type x Upper tempo x Region 3 | 12.386 | 10.608 | 1179.83 | 1.168 | 0.243 | -- |
| Trial type x Lower tempo x Region 3 | -7.861 | 9.154 | 1179.83 | -0.859 | 0.391 | -- |
| Music exp. x Upper tempo x Region 2 | 6.461 | 10.608 | 1179.83 | 0.609 | 0.543 | -- |
| Music exp. x Lower tempo x Region 2 | -7.528 | 9.154 | 1179.83 | -0.822 | 0.411 | -- |
| Music exp. x Upper tempo x Region 3 | 5.872 | 10.608 | 1179.83 | 0.554 | 0.580 | -- |
| Music exp. x Lower tempo x Region 3 | -7.756 | 9.154 | 1179.83 | -0.847 | 0.397 | -- |
| Trial type x Music exp. x Upper tem. x Reg. 2 | 2.969 | 21.216 | 1179.83 | 0.14 | 0.889 | -- |
| Trial type x Music exp. x Lower tem. x Reg. 2 | -6.305 | 18.309 | 1179.83 | -0.344 | 0.731 | -- |
| Trial type x Music exp. x Upper tem. x Reg. 3 | 2.431 | 21.216 | 1179.83 | 0.115 | 0.909 | -- |
| Trial type x Music exp. x Lower tem. x Reg. 3 | -21.194 | 18.309 | 1179.83 | -1.158 | 0.247 | -- |
| **MAXL** *Marginal R2 = 0.180, Conditional R2 = 0.320* | | | | | | |
| Predictors | Estimate | SE | *df* | *t-value* | *p-value* | *ß* |
| (Intercept) | 0.982 | 0.101 | 23.25 | 9.701 | < 0.0001 | -- |
| Trial type | -0.739 | 0.068 | 1202.50 | -10.792 | < 0.0001 | -.33 *** |
| Music experience | -0.092 | 0.202 | 23.25 | -0.454 | 0.654 | -- |
| Region 2 | -0.039 | 0.078 | 1179.95 | -0.504 | 0.614 | -- |
| Region 3 | -0.323 | 0.078 | 1179.95 | -4.134 | < 0.0001 | -.14 *** |
| Upper tempo range | -0.076 | 0.080 | 1185.30 | -0.95 | 0.342 | -- |
| Lower tempo range | -0.253 | 0.069 | 1184.46 | -3.664 | < 0.0001 | -.11 *** |
| Trial type x Music experience | -0.131 | 0.137 | 1202.50 | -0.955 | 0.340 | -- |
| Trial type x Upper tempo range | -0.240 | 0.162 | 1187.15 | -1.489 | 0.137 | -- |
| Trial type x Lower tempo range | 0.016 | 0.141 | 1192.49 | 0.11 | 0.912 | -- |
| Music experience x Upper tempo | -0.061 | 0.161 | 1185.30 | -0.376 | 0.707 | -- |
| Music experience x Lower tempo | -0.017 | 0.138 | 1184.46 | -0.124 | 0.901 | -- |
| Trial type x Region 2 | -0.182 | 0.156 | 1179.95 | -1.166 | 0.244 | -- |
| Trial type x Region 3 | 0.375 | 0.156 | 1179.95 | 2.397 | 0.017 | .08 * |
| Music experience x Region 2 | -0.191 | 0.156 | 1179.95 | -1.221 | 0.222 | -- |
| Music experience x Region 3 | 0.134 | 0.156 | 1179.95 | 0.855 | 0.393 | -- |
| Upper tempo x Region 2 | -0.274 | 0.195 | 1179.95 | -1.405 | 0.160 | -- |
| Lower tempo x Region 2 | -0.099 | 0.168 | 1179.95 | -0.586 | 0.558 | -- |
| Upper tempo x Region 3 | -0.326 | 0.195 | 1179.95 | -1.674 | 0.094 | -.06 . |
| Lower tempo x Region 3 | 0.198 | 0.168 | 1179.95 | 1.178 | 0.239 | -- |
| Trial type x Music exp. x Upper tempo | -0.505 | 0.323 | 1187.15 | -1.564 | 0.118 | -- |
| Trial type x Music exp. x Lower tempo | 0.113 | 0.283 | 1192.49 | 0.401 | 0.688 | -- |
| Trial type x Music exp. x Region 2 | 0.436 | 0.312 | 1179.95 | 1.397 | 0.163 | -- |
| Trial type x Music exp. x Region 3 | -0.079 | 0.312 | 1179.95 | -0.253 | 0.800 | -- |
| Trial type x Upper tempo x Region 2 | 0.174 | 0.390 | 1179.95 | 0.445 | 0.656 | -- |
| Trial type x Lower tempo x Region 2 | 0.230 | 0.336 | 1179.95 | 0.682 | 0.495 | -- |
| Trial type x Upper tempo x Region 3 | 0.615 | 0.390 | 1179.95 | 1.578 | 0.115 | -- |
| Trial type x Lower tempo x Region 3 | -0.393 | 0.336 | 1179.95 | -1.169 | 0.243 | -- |
| Music exp. x Upper tempo x Region 2 | -0.143 | 0.390 | 1179.95 | -0.367 | 0.713 | -- |
| Music exp. x Lower tempo x Region 2 | -0.065 | 0.336 | 1179.95 | -0.193 | 0.847 | -- |
| Music exp. x Upper tempo x Region 3 | -0.323 | 0.390 | 1179.95 | -0.83 | 0.407 | -- |
| Music exp. x Lower tempo x Region 3 | -0.323 | 0.336 | 1179.95 | -0.959 | 0.338 | -- |
| Trial type x Music exp. x Upper tem. x Reg. 2 | 0.771 | 0.780 | 1179.95 | 0.989 | 0.323 | -- |
| Trial type x Music exp. x Lower tem. x Reg. 2 | -0.149 | 0.673 | 1179.95 | -0.222 | 0.824 | -- |
| Trial type x Music exp. x Upper tem. x Reg. 3 | 0.568 | 0.780 | 1179.95 | 0.729 | 0.466 | -- |
| Trial type x Music exp. x Lower tem. x Reg. 3 | -0.732 | 0.673 | 1179.95 | -1.089 | 0.277 | -- |
