# Supplementary material for: Multidimensional recurrence quantification analysis of human-metronome phasing
Source: PLoS One. 2023 Feb 23;18(2):e0279987. doi: 10.1371/journal.pone.0279987 (PMC9949643; doi:10.1371/journal.pone.0279987)
Supplement: S3 Appendix — (DOCX) [file pone.0279987.s003.docx]

This coding scheme yields *k*-1 variables to account for each factor of *k* levels, each testing the difference between a given level and the reference level. For general MdRQA, this yielded the following variables: multilingual participants (as compared against monolingual participants) = 1/2 (multilingual) or -1/2 (monolingual); lower-range tempi (as compared against middle-range tempi) = 2/3 (lower-range tempi) or -1/3 (all other tempi); upper-range tempi (as compared against middle-range tempi) = 2/3 (upper-range tempi) or -1/3 (all other tempi); successful trials (as compared with incomplete trials) = 2/3 (successful trials) or -1/3 (all other trial types); unsuccessful trials (as compared with incomplete trials) = 2/3 (unsuccessful trials) or -1/3 (all other trial types).
For region-based MdRQA, this yielded the following variables: multilingual participants (as compared against monolingual participants) = 1/2 (multilingual) or -1/2 (monolingual); lower-range tempi (as compared against middle-range tempi) = 2/3 (lower-range tempi) or -1/3 (all other tempi); upper-range tempi (as compared against middle-range tempi) = 2/3 (upper-range tempi) or -1/3 (all other tempi); unsuccessful trials (as compared against successful trials) = 1/2 (unsuccessful trials) or -1/2 (successful trials); region 2 (as compared against region 1) = 2/3 (region 2) or -1/3 (all other regions); region 3 (as compared against region 1) = 2/3 (region 3) or -1/3 (all other regions).
