# Supplementary material for: Multidimensional recurrence quantification analysis of human-metronome phasing
Source: PLoS One. 2023 Feb 23;18(2):e0279987. doi: 10.1371/journal.pone.0279987 (PMC9949643; doi:10.1371/journal.pone.0279987)
Supplement: Attachment — Submitted filename: PLOS One Response to Reviewers.docx [file pone.0279987.s004.docx]

***Journal Requirements***

—

1. Please ensure that your manuscript meets PLOS ONE's style requirements, including those

for file naming. The PLOS ONE style templates can be found at

https://journals.plos.org/plosone/s/file?id=wjVg/PLOSOne_formatting_sample_main_body.pdf

and

https://journals.plos.org/plosone/s/file?id=ba62/PLOSOne_formatting_sample_

title_authors_affiliations.pdf.

*We have updated our manuscript’s format to adhere to PLOS ONE’s style requirements.*

2. Please change "female” or "male" to "woman” or "man" as appropriate, when used as a

noun (see for instance https://apastyle.apa.org/style-grammar-guidelines/bias-freelanguage/

gender).

*The correct wording is “female” and “male” in this case because our survey asked participants their sex.*

3. Thank you for stating the following in the Competing Interests/Financial Disclosure:

I have read the journal's policy and the authors of this manuscript have the following

competing interests: EWL holds ownership interest in Oscilloscape, LLC.

We note that one or more of the authors are employed by a commercial company: EWL holds

ownership interest in Oscilloscape, LLC.

a. Please provide an amended Funding Statement declaring this commercial affiliation, as well

as a statement regarding the Role of Funders in your study. If the funding organization did not

play a role in the study design, data collection and analysis, decision to publish, or preparation

of the manuscript and only provided financial support in the form of authors' salaries and/or

research materials, please review your statements relating to the author contributions, and

ensure you have specifically and accurately indicated the role(s) that these authors had in your

study. You can update author roles in the Author Contributions section of the online submission

form.

Please also include the following statement within your amended Funding Statement.

“The funder provided support in the form of salaries for authors [insert relevant initials], but did

not have any additional role in the study design, data collection and analysis, decision to

publish, or preparation of the manuscript. The specific roles of these authors are articulated in

the ‘author contributions’ section.”

If your commercial affiliation did play a role in your study, please state and explain this role

within your updated Funding Statement.

b. Please also provide an updated Competing Interests Statement declaring this commercial

affiliation along with any other relevant declarations relating to employment, consultancy,

patents, products in development, or marketed products, etc.

Within your Competing Interests Statement, please confirm that this commercial affiliation

does not alter your adherence to all PLOS ONE policies on sharing data and materials by

including the following statement: ""This does not alter our adherence to PLOS ONE policies

on sharing data and materials.” (as detailed online in our guide for authors

http://journals.plos.org/plosone/s/competing-interests) . If this adherence statement is not

accurate and there are restrictions on sharing of data and/or materials, please state these.

Please note that we cannot proceed with consideration of your article until this information has

been declared.

Please include both an updated Funding Statement and Competing Interests Statement in

your cover letter. We will change the online submission form on your behalf.

*Edward Large has been removed as an author, and thus there is no longer a competing interest. We have removed the statement entirely.*

4. We note that Figure 2 in your submission contain copyrighted images. All PLOS content is

published under the Creative Commons Attribution License (CC BY 4.0), which means that the

manuscript, images, and Supporting Information files will be freely available online, and any

third party is permitted to access, download, copy, distribute, and use these materials in any

way, even commercially, with proper attribution. For more information, see our copyright

guidelines: http://journals.plos.org/plosone/s/licenses-and-copyright.

We require you to either (1) present written permission from the copyright holder to publish

these figures specifically under the CC BY 4.0 license, or (2) remove the figures from your

submission:

a. You may seek permission from the original copyright holder of Figure 2 to publish the

content specifically under the CC BY 4.0 license.

We recommend that you contact the original copyright holder with the Content Permission

Form (http://journals.plos.org/plosone/s/file?id=7c09/content-permission-form.pdf) and the

following text:

“I request permission for the open-access journal PLOS ONE to publish XXX under the

Creative Commons Attribution License (CCAL) CC BY 4.0 (http://creativecommons.org/

licenses/by/4.0/). Please be aware that this license allows unrestricted use and distribution,

even commercially, by third parties. Please reply and provide explicit written permission to

publish XXX under a CC BY license and complete the attached form.”

Please upload the completed Content Permission Form or other proof of granted permissions

as an ""Other"" file with your submission.

In the figure caption of the copyrighted figure, please include the following text: “Reprinted

from [ref] under a CC BY license, with permission from [name of publisher], original copyright

[original copyright year].”

b. If you are unable to obtain permission from the original copyright holder to publish these

figures under the CC BY 4.0 license or if the copyright holder’s requirements are incompatible

with the CC BY 4.0 license, please either i) remove the figure or ii) supply a replacement figure

that complies with the CC BY 4.0 license. Please check copyright information on all

replacement figures and update the figure caption with source information. If applicable,

please specify in the figure caption text when a figure is similar but not identical to the original

image and is therefore for illustrative purposes only.

*Michael Schutz, the publisher of the original figure, gave us written permission to reproduce Fig 1 via email on January 29, 2021. The email correspondence is uploaded as the file titled “Other.” We added “(Reprinted from [4] under a CC BY license, with permission from Michael Schutz, original copyright 2019.)” to the caption of figure 1.*

5. Please include a caption for figure 8 in manuscript.

*Figure 8 is no longer included in the manuscript. All other figure captions are now included.*

***General comment to reviewers****: In addressing the reviewers’ comments, we noticed an error in our coding for categorical variables. We have corrected the error and have adjusted the text and tables accordingly.*

—

**Reviewer #1**: The current study aims to test the roles of multilingualism and tempo in predicting perception-action coordination. To evaluate these effects, the authors conducted a phasing-based experiment – tapping a tempo deviated from a specific BPM played by a metronome. Innovatively, the sample did not consist of professional musicians. Due to better selective attention, the researchers hypothesized that multilingual participants would show higher rates of success in the task – defined as a return to in-phase tap after one lap of desynchronization. Further, they assessed that around both antiphase and in-phase subjects would tap more consistently, and that 100-120 BPM would be the most compatible tempi to succeed in phasing. The reasoning to hypothesize the latter was vague.

Multi-dimensional Recurrence Quantification Analysis was applied on components of the relative phase between participants' taps and original tempo set by the metronome. In this manner, the researchers evaluated several aspects of the performance on the task. Their findings did not support the hypothesis regarding the suitable tempo for phasing. Instead, they found that participants are drawn more to a specific relative phase as the tempo increases from 80 to 140 BPM. Windowed mdRQA implied that in-phase is a stronger attractor than antiphase on trials with higher tempi. While monolinguals’ transition in and out of synchrony is affected by tempo, multilingual subjects could keep a stable tapping rhythm across the range of BPM – corresponding with the authors' hypothesis. However, a variance test was not provided.

***Response****: We agree with the reviewer’s concern and have clarified the intention of our hypothesis in the current version. We were intending this to capture the noise of the system through %REC; as such, we changed language to “less noisy” instead of “more stable.”*

The paradigm was based on previous research and fitted to non-musicians, hence helping to generalize Schutz's previous findings regarding phasing. The authors declared their hypotheses precisely and returned to them in the discussion section. Also, Recurrence Quantification Analysis was an innovative and suitable way to approach this data – supported by the researchers' findings of higher mdRQA attributes on successful trials. Moreover, the supplemented data, as well as the videos, and codes were plain and explanatory.

My primary concerns are:

• The reasoning behind conducting mdRQA on the sine and cosine of the relative phase is unclear. How should a recurrence point of cosine and sine be interpreted?

- ***Response****: We apologize for the lack of clarity in the previous version and have attempted to add more detail about this to the revision (****pp. 4-6, “Quantifying Phasing with a Novel Application of RQA”; pp. 10-13, “General MdRQA” and “Windowed MdRQA”****).

  We used MdRQA over the decomposed circular relative phase values because there is no circular version of RQA. This is important because of the* seeming *discontinuity in numbers in angles between 359 and 0; while we can visually see that the differences between those numbers are the same as the difference between 179 and 180, standard versions of RQA would see them as quite different.

  We chose MdRQA rather than CRQA because the x- and y-values of the circular relative phase comprise a multivariate measurement of the same human-metronome system. These data are therefore more consistent with the principles of MdRQA (which captures the dynamics of a single multidimensional system) rather than those of CRQA (which captures the shared trajectories of two univariate systems). While CRQA would be appropriate for comparing ticks of the metronome with taps of the participant (i.e., two univariate trajectories), the decomposition of the relative phase value into two component parts would still be two signals from the same system. However, simply conducting categorical CRQA on the metronome ticks and participant taps would not uncover the richness of the relative phase relation that we hope to uncover, especially under the relatively short time series under consideration.

  With MdRQA, we have a two-dimensional vector, including the x- and y-coordinates of each circular relative phase value.*

• The windowed analysis’s relatively high SD for the mdRQA outcomes (REC, DET, maxL) suggests that the time series may be too short to conduct mdRQA properly. To address this point, the authors should check the average size of the recurrence plots and the histogram of the mdRQA outcomes.

- ***Response****: According to Wallot and Leonardi (2018), MdRQA can be properly conducted with as few as 10-30 data points. On average, our recurrence plots contain 18.6 data points in each phasing window. In keeping with open science principles, we include all data in our OSF repository to provide maximal transparency. For clarity with the reviewers, we have included histograms of the outcomes—for all data in both general and windowed MdRQA—below.*

• The researchers should provide the mdRQA parameters that were used for the analysis.

- ***Response****: The MdRQA parameters can be found in the MATLAB code posted on OSF (link included on* ***p. 10, “Open Materials, Data, and Code”****). We thank you for pointing out that these should be included in the main text, and we agree the analysis would be clearer that way.*

*We added the parameter values along with an explanation for why each was chosen in the “Data Analysis” section (****p. 12, “General MdRQA”****). The 5 parameters we used in general and windowed MdRQA included number of embedding dimensions (EMB = 1), delay (DEL = 1), type of norm by which the phase space is normalized (NORM = “non”), radius size (RAD = 1), and whether the data should be z-scored (ZSCORE = 0).*

*Further detail in “Data Analysis” describes why we used unembedded times series with no time-delay, why we did not normalize or reconstruct the phase space, why a radius of one is appropriate in this case, and why we did not z-score out data.*

My minor concerns are:

• Effect size would be an essential addition to the Tukey posthoc, specifically when the degrees of freedom are high.

- ***Response****: Thank you for pointing this out. We added columns reflecting standardized effect sizes (****Table 2, Table 4****).*

• In my opinion, a recurrence point should not be defined as a repetition of the same value. In this case, it seems that a recurrence point stands for similarity dependent on the chosen radius.

- ***Response****: Thank you for noticing this. We changed the definition of a recurrence point to reflect similarity within a given radius (****p. 12, “General MdRQA”****).*

• Why does mdRQA fit this data better than CRQA?

- ***Response****: CRQA measures the coevolution of two distinct time series. MdRQA quantifies the auto-recurrence properties of a single multidimensional time series. In our analysis, each trial has only one time series (i.e., relative phase). We decompose relative phase into two dimensions: x- and y-coordinates of circular relative phase values. Therefore, each point on the recurrence plot contains a two-dimensional vector of relative phase, and this time series is plotted on both axes (****pp. 11-12, “General MdRQA”****).*

If the authors address the above queries, I think the paper could contribute to the literature.

***Response****: In addition to the above changes outlined, we addressed your comment about the unclear reasoning behind the hypothesis that 100-120 BPM would be the most compatible tempi to succeed in phasing. This prediction aligns with the tapping literature on preferred tempo (p. 7, “Present Study”).*

**Reviewer #2**:

Comments to the editor and authors:

I would like to thank the authors for the opportunity to read this manuscript. The paper reports an empirical study of a ‘phasing’ task using multidimensional recurrence quantification analysis. While I appreciate the effort put in this work, the manuscript has major problems and the overall quality is far below adequate for publication. In brief, the place of this study within past research is not well established, the task is not described in enough detail, the analyses (MdRQA, regression, and posthoc tests) are not presented in enough detail, the results are not properly reported and interpreted, and some of the analyses reported are not appropriate to assess the hypotheses put forward. In addition, the text is unclear in many passages, and the limitations are not addressed.

Therefore, I have no alternative than to recommend rejecting this submission.

It would be impractical to list all problems I found in this manuscript. Below I will list some of the main issues. I hope the authors will see my comments are meant to help them improve the manuscript for a future submission.

L75ff: about RQA

The analytical framework of RQA is presented very superficially as a method “to describe nonlinearities of coupled dynamical systems”. The reader must be presented with a bit more of the conceptual (if not mathematical) background.

- ***Response****: We apologize for the vague description of RQA, and we agree that more detail would help readers better understand the paper overall. We added more conceptual background of RQA and its various extensions under the “RQA” heading. We also referred readers to literature containing in-depth mathematical explanations of RQA and its extensions (****pp. 4-6, “Quantifying Phasing with a Novel Application of RQA”****).*

L76ff: about MdRQA.

Wallot et al (2016) present MdRQA as a method to “analyze group-level behavior of groups bigger than a dyad.” Given the current study design can be conceived of as involving human-metronome 'dyads', you must explain better why you chose MdRQA. Further below in the text you indicate that using MdRQA was not the initial choice but an ad hoc decision, a workaround because of issues with the data. This must be made clear.

- ***Response:*** *We realize that the description of our motivation for selecting MdRQA was too vague in the previous version of our manuscript. As noted in our response to Reviewer 1, we have added substantial detail about this decision in the revised version (****pp. 4-6, “Quantifying Phasing with a Novel Application of RQA”; pp. 11-13, “General MdRQA” and “Windowed MdRQA”****).*

*To clarify further, while MdRQA can be used on data that is bigger than a dyad, it can be used with as few as two variables. Wallot et al. (2016) gives an example of this in the “Example: Origami Production Task” section.

We have also attempted to more clearly articulate the changes in our analysis plan so that it does not appear as though MdRQA was our original intention (****p. 6, “Quantifying Phasing with a Novel Application of RQA”****). As a preliminary experimental study of this phenomenon, we agree about the importance of being especially clear about our analysis choices.*

L72: “The “static” part actually varies along with the moving part…”

This is too vague. What is it that you want the reader to attend?

- ***Response****: The drummer who intended to maintain the original tempo (i.e., “static part”) was unable to do so, and instead increased and decreased their tempo along with the phasing drummer (i.e., “moving part”). We added more clarity to the Figure 1 caption (****p. 4****).*

L85:

Calling 25 WEIRD undergraduates with age range 18-21 “a broad population… with different musical and linguistic backgrounds” is not realistic.

- ***Response****: This is a good point, thank you. We changed “broad population” to “a population with more varied musical and linguistic backgrounds” (****p. 6, “Present Study”****).*

L92ff Hypotheses

1) Please indicate your hypotheses and the predictions tested more clearly. I identified 3 (or perhaps 4) hypotheses/predictions:

H1: “participants will demonstrate stable tapping near in-phase and antiphase” with prediction “higher metrics during those periods.”

To test H1, I suppose one would compare inphase and antiphase, which seems related to the windowed analysis, but this is unclear. The motivation to conduct windowed MdRQA is said to be “to compare successful and unsuccessful trials” so the analytical framework and the implications for the hypothesis are confusing.

H2A: “multilingual participants may be able to simultaneously attend to the metronome while adopting a different tempo.” H2B: “Monolingual participants may experience stronger coupling with the metronome” with prediction: “greater metrics for monolingual speakers.”

To test H2, the idea seems to be to use regression analysis, but since the interactions are not properly taken into account in the report, you end up not able to compare between monolingual and multilingual participants appropriately.

Also, given you simplified the task, the prediction in L102 does not seem well justified.

H3: “we expect the middle range of our selected tempi (100-120 bpm) to yield the most structured phasing performance for all participants” with prediction “higher metrics”

To test H3, I suppose one would compare middle range with lower (80-100) and higher (120-140) ranges. But in this case, adding ‘tempo’ as a linear predictor in the models does not seem to be the appropriate way to test this. Because your prediction is that the relationship is not linear but perhaps something like parabolic: low-high-low

2) How does comparing successful and unsuccessful trials relate to the hypotheses presented? In other words, why did you add trial type as a predictor in the models?

- ***Response****: Thank you for the helpful suggestions for how to outline our hypotheses. We added a subsection to the introduction called “Hypotheses” where we write each of our predictions in a numbered list (****pp. 6-8, “Hypotheses”****). Below is clarification for each of your questions/comments:*
  - *1) Hypotheses*
    - *H1: You are correct that H1 is to compare phase relationships using windowed MdRQA. We apologize for the lack of clarity on how this was done. We added more detail about windowed MdRQA and why it’s suited to test this hypothesis in the “Data Analysis” section (****pp. 11-13, “General MdRQA” and “Windowed MdRQA”****).*
    - *H2: This hypothesis was tested using a linear mixed-effects model, and we have provided additional clarifications about the model structure in our response to questions about interaction terms (see response to comment about “L141ff Data analysis,” below). We added further explanation in “Results and Discussion” (****pp. 14-18****) to more accurately reflect the fact that all interactions were included in the original model.*
    - *H3: You make a great point about the linear model not being suited to test H3. We updated our test of H3 by binning tempi into lower (80-90 bpm), middle (100-120 bpm), and upper (130-140 bpm) ranges. Thank you for your suggestion.*
  - *2) Comparing Successful and Unsuccessful Trials was an exploratory analysis, as we did not anticipate the high percentage of participants who wouldn’t be able to complete the simplified phasing task. We did not have specific predictions regarding Successful and Unsuccessful Trials. Trial type was added as a predictor because we wanted to compare the performance of those who could and couldn’t complete the phasing task. This allowed us to investigate how the underlying dynamics compare to Schutz’s case for both trial types.*
- ***Note****: To improve flow, we reordered the hypotheses. H1 from the original manuscript is now H2 in the revised version, H2 from the original is H3 in the revised version, and H3 from the original is H1 in the revised version.*

L112: sample size

What you report is not what is commonly meant by 'determining sample size'. What exactly was the sample size determined in advance? Anything from 15 – 30? Which 'groups' did you consider relevant to determine sample size? Considering the vars gender (male/female), linguistic abilities (mono/multi), and musical experience (yes/no), you do not have 15-30 in each group.

- ***Response****: We recruited 25 non-expert participants for an exploratory study. We did not recruit based on planned group comparison (****p. 8, “Participants”****). We have further clarified that we did not include gender as a covariate in our* a priori *expectations or any analyses.*

L115 Data inclusion criteria and n of trials

Given you need to know what a trial is to understand this part, I suggest you move it to after you describe the procedure, i.e. before Data Analysis.

- ***Response****: Thank you for this suggestion; we agree that rearranging the sections would improve readers’ understanding. We moved “Procedure” before “Data Inclusion Criteria” to account for this.*

L115

Please justify the number of trials in the dataset. What counts as a trial, how many trials per participant, how many issues e.g. due to early termination, malfunction?

- ***Response****: We have moved a significant amount of the method from our supplementary materials and into the body of the manuscript in order to improve clarity. One trial consists of the following phasing method: Participants started the trial tapping in synchrony with the metronome for several beats. At the sound of a warning signal, they began phasing by increasing their tempo slightly and then maintaining that tempo until synchronized with the metronome again. Resynchronization happens naturally because of the tempo difference. Once resynchronized, participants were instructed to tap at the metronome’s tempo again for several beats and then stop. A single trial lasted a maximum of two minutes but took less time the faster participants phased. This procedure is described in more detail in “Procedure” in the main text (****pp. 8-10****) and in S1 Appendix. The number of trials is justified in “Data Inclusion Criteria” (****p. 10****).*

L116 , then L137

Please clarify what you mean by ‘one round of phasing’ and “complete one phasing lap”. The whole procedure is a bit unclear for the reader

- ***Response****: This is specified in S1 Appendix. To make the main text clearer, we defined one phasing lap as “The period between participants’ initial desynchronization with the metronome and their resynchronization after increasing their tempo” (****p. 9, “Procedure”****).*

L119

Given the instructions to participants, how can you not have any tapping data in some trials? What is the justification to recode these missing data as zero? If this was done merely to be able to fit the models, this is probably wrong.

- ***Response****: Thank you for bringing our attention to this point. In the original manuscript, it was an error to state that trials with no tapping data were assigned values of zero for all MdRQA metrics. Only one of the trials lacked tapping data, and this trial was excluded from our analyses, not assigned a value of zero. For the rest of the trials, even Incomplete Trials, there is tapping data and nothing is assigned a value of zero. We added this clarification under “Data Inclusion Criteria” (****p. 10****).*

L131

You mention demographics survey and then do not report/discuss anything. What exactly do you mean here?

- ***Response****: Music and linguistic experience were the only factors from the demographics survey used in the analyses. (That is, we did not plan to analyze them when the study was designed, nor did we conduct analyses on them later.) All other demographic information was collected for IRB purposes. We added this clarification under “Procedure” (****p. 7****).*

L141ff Data analysis

1) You must report the parameters used in MdRQA and how you decided which values to use.

2) The Statistical analyses conducted must be properly described. Simply saying that you calculated descriptive and inferential statistics is too vague. You fitted regression models and you must describe what you did in detail so that readers can understand and assess your work. The structure of the models fitted must be clearly explained. For example, the tables suggests you included interactions and you did not mention that. Describe each predictor, their possible values and scale. Were they centered or standardized prior to including in the models? How did you account for repeated measures arising from multiple trials? The [ ] notation in the table suggests you used 'random' effects, if so what and why and how? One table indicates you added a 4-way interaction: trialtype*language*tempo*window but not all parameter combinations seem present. As the moment it is not at all clear that the regression models were structured (declared) properly and I have very low confidence in the reported results and their interpretation. Also, Table 5 reports post hoc tests but you did not clearly explain them.

3) As it is, the text is saying you used all these R packages to calculate statistics, which is not the case (e.g. viridis). If you indeed want to acknowledge all packages you used, you must clarify their role in the analyses.

- ***Response****:*

*1) As explained above in our response to Reviewer 1, parameters are specified in our publicly available code, and we added the parameter values and how they were selected to “General MdRQA” (****p. 11****).*

*2) This is a great point, thank you for the suggestion. We added a section called “Model Specifications” in which we go into detail about our linear mixed-effects models and post-hoc tests (****p. 13****). We apologize for any lack of clarity in our previous version: Because Successful Trials, monolingual language experience, and window 1 are used as the baseline in the model (i.e., reference categories), all parameter combinations in Table 4 (i.e., the 4-way interaction) are represented. We added this clarification to “Results and Discussion” (****p. 13****).*

*3) This is a great point, thank you. We changed the text to reflect that we used these packages for data manipulation, visualization, and analysis, with specific implementation of each included in the open project code (****p. 10****).*

L146

Please add at least a sentence to clarify to the reader what phi is conceptually (relative phase between participants’ taps and metronome ticks). Please format the formula correctly to improve readability.

- ***Response****: Phi is relative phase, which measures the difference between the time at which a participant taps and the nearest metronome tick, yielding the phasing relationship between participant and metronome. We added this description and reformatted the equation in “Relative Phase Calculation” (****p. 9****).*

L154

What do you mean by 'the apparent discontinuity in Ψ near synchrony'?

- ***Response****: There is a* seeming *discontinuity in numbers in angles between 359 and 0; while we can visually see that the differences between those numbers are the same as the difference between 179 and 180, standard versions of RQA would see them as quite different. We added more detail to “Quantifying Phasing with a Novel Application of RQA” (****p. 5****) and to “General MdRQA” to better explain this (****p. 11****).*

L154

1)The procedure to ‘adapt’ the data for MdRQA must be better motivated and better explained (to say you “decomposed each tap into its x- and y-coordinates by taking cosine and sine” is not very clear).

2)In what ways does the procedure adapt the data to mdrqa? How did this solve the problem of 'apparent discontinuity'?

***Response****: We apologize for the lack of detail here and agree that our motivation and implementation of MdRQA should be better explained. Similar questions were asked by Reviewer 1 (see above), and we have worked to address them in our manuscript. While more details are provided in our response to Reviewer 1, we note here that we added in-depth explanations under “General MdRQA” (****p. 11)****.*

L162 windowed analysis

1)please clarify the motivation to conduct windowed analysis: is this related to H1? How does this relate to comparing successful and unsuccessful trials?

2)Did you identify the transitions from one window to another for each time series? How?

3)Fig 2: The figure does not show MdRQA but a MdR plot. Why is the in-phase period not represented in this MdRplot?

4) Fig 2: caption is incorrect. The time series may be perhaps 'represented' in both axes, but this plot shows recurrences not the time series. The axes labels need adjusting as does the title.

- ***Response****:*

*1) We added a brief introduction to windowed MdRQA to the “RQA” section (****p. 5****) and to “Windowed MdRQ” (****p. 11-12****). We used windowed MdRQA to compare different phasing relationships in the human-metronome system (e.g., synchrony, antiphase). This was done to address H1 and in our exploratory analyses of Successful and Unsuccessful Trials. We did not have* a priori *hypotheses regarding Successful versus Unsuccessful Trials because we did not anticipate the emergence of Unsuccessful trials, but we used windowed MdMQA to assess how those who could and couldn’t complete the task compared to the original case study of phasing.*

*2) Transitions are naturally taken into account by the progression from each window. Our analyses segmented the relative phase relations into the following discrete and non-overlapping regions: 45° ≤ 𝛹 < 135° were window 1, 135° ≤ 𝛹 < 225° were window 2, and 225° ≤ 𝛹 < 315°. This description appears under “Windowed MdRQA” in our updated manuscript (****pp. 11-12****) and is depicted in Figure 2B.*

*3) We have attempted to clarify this in our caption. We have updated the caption of Figure 2 to the following: “Sample multidimensional recurrence plot (MdRP) produced for one phasing trial. [...] (B) [...] As described in “Windowed MdRQA,” synchronous taps were excluded from windowed MdRQA to effectively compare Successful and Unsuccessful Trials” (****pp. 12-13****).*

*4) We have updated the caption as follows: “(A) How subsections were created from the MdRP to conduct windowed analysis. Each sample in the time series contains a vector of x- and y-coordinates of 𝛹. Recurrences or revisitations of the same multidimensional space (within a given tolerance) between the two time series (i.e., the same time series represented on the x-axis and y-axis) are plotted as points on the MdRP” (****pp. 12-13****).*

L177:

Certainly not “all results”, so please clarify which results do you report (e.g. summary statistics, table of estimates from regression models). You should also consider reporting some effect sizes (e.g. relevant slopes) in the text if you want to use them to support your inferences.

- ***Response****: We changed “all results” to “Statistically significant and nonsignificant results are presented in tables; only statistically significant results (p < 0.05) are discussed in the text” in “Results and “Discussion” (****p. 14****). We agree about the importance of reporting effect sizes and apologize for this shortcoming in our original manuscript. For readability and clarity within the text, effect sizes have been added to the tables but not reiterated in the main text.*

L184 Fig 3

Table 2 suggests the model included interactions. But Fig 3 shows raw data and does not take interactions into account. Also, what does the error bar represent?

A general note: The tables are poorly formatted and take too much space.

- ***Response:*** *We appreciate the reviewer’s concerns about clarity in our figures and tables. In order to maximize readability, we have clarified the row names in the tables to make them more reader-friendly. We understand the reviewer’s concern about the number of tables, but we have chosen to present complete results for all models run in the project in order to provide as comprehensive a record as possible of the present work. We adjusted figures 3-5 to depict interaction effects.*

L213 “significant interaction for tempo, window, and trial type”

1) Please clarify this passage, as it is not simply 'the interaction' that is relevant here but the estimated effects. Also, it is incorrect to say interaction ‘for’ A, B, and C in this context.

2) in what ways did the results “conceptually replicate” previous findings?

- ***Response****:*

*1) Thank you for pointing out this mistake. We changed “interaction for” to “interaction among” or “interaction between” (****pp. 15-18****). Additionally, we added the estimated effect sizes for this finding to Table 4.*

*2) The conceptual replication is the finding that the two stable phase relationships are synchrony and antiphase, which is what we see in the tapping literature. Stable phase relationships were demonstrated by higher metrics in windows 1 and 2 than in window 3, which may be related to the differential pulls toward synchrony and antiphase. More detail can be found in “Results and Discussion” (****p. 14****).*

L214

what do you mean by ‘relative stability’?

- ***Response****: We mean that the tapping pattern has a stronger attractor during windows 1 and 2 as compared to window 3. “Relative stability” is indicated by an increased number of consecutive recurrent states (i.e., greater maxline value). We added clarification to our description of relative stability in our revision (****p. 16****).*

L229: “our finding that inhibitory control varies based on language experience and tempo”

Which results specifically do you think supports your claim, please report the relevant estimates, effect sizes, and statistics.

- ***Response****: After re-running our analyses--this time using contrast coding--we discovered that it’s not as straightforward as our previous interpretation that “inhibitory control varies based on language experience and tempo.” We found a significant interaction effect between language experience and tempo on %REC and significant interaction effects among language expereince, tempo, and window on %DET and MAXL. Altogether, these findings suggest a complex relationship between task parameters (i.e., tempo), language experience, and phase relationship. Detailed interpretations of each significant interaction can be found in the “Hypothesis 3” subsection of “Results and Discussion” (****pp. 17-18****). Estimates, effect sizes, and statistics are in the tables.*

L289 Conclusion

It is not clear that your results provided a 'broad replication of the original dynamics' reported by Schultz.

- ***Response****: We have attempted to more clearly describe what we mean by this claim in our revision. Participants from Successful Trials broadly replicate the original dynamics by dwelling near in-phase and antiphase and quickly transitioning between these attractors, regardless of musical experience, tempo, or task demands. This pattern was first shown in Schutz’s case study in an expert sample and with different task demands. This suggests similar sensorimotor coordination processes may underlie successful phasing, even for different populations performing at different rhythmic complexities (e.g., isochronous versus non-isochronous). We added these details to “Conclusion” (****p. 20****).*

L290 “sensorimotor coordination processes may underlie successful phasing”

what would it mean to say that sensorimotor coordination processes did NOT underlie successful phasing?

- ***Response****: Thank you for directing us to clarify this distinction, as it is very important to our overall findings. What we mean by this phrase is that phasing may be more related to underlying SMS abilities than to musical expertise or rhythmic complexity. This is supported by the similarity in dynamics between the professional percussionists from Schutz’s case study and our sample of non-experts with varying musical and linguistic backgrounds. Unsuccessful Trials, on the other hand, did not mirror the dynamics from the case study, which is why we claimed that similar sensorimotor coordination processes may underlie* ***successful*** *phasing (as opposed to unsuccessful). We added more detail to “Conclusion” (****p. 20****).*

A general comment about the figures

Please indicate clearly what the error bars show. The titles of figures must be revised to correct typos, grammar mistakes and to improve their meaning, especially figures 2,4, 5, and 6. For ex, it makes no sense to say ‘interaction between language experience, window, and tempo on DET’

A general comment about openly available data

Please consider making the data available in a more friendly format such as a csv or txt, as matlab is perhaps too restrictive.

- ***Response****: Thank you for this suggestion. We included the data as a csv file in our OSF page (https://osf.io/4uhtb/?view_only=f8b5a974b1b54bff95abbc41a8342f5e).*
