# Supplementary material for: Multidimensional recurrence quantification analysis of human-metronome phasing
Source: PLoS One. 2023 Feb 23;18(2):e0279987. doi: 10.1371/journal.pone.0279987 (PMC9949643; doi:10.1371/journal.pone.0279987)
Supplement: Attachment — Submitted filename: 10_18_22 response to reviewers.docx [file pone.0279987.s005.docx]

Reviewer #1: The authors improved the paper and responded thoroughly to the points of

concern. In my opinion, the manuscript can now be accepted.

Reviewer #2: My recommendation is major revision.

Thank you for the opportunity to revisit this manuscript. I appreciate the effort put by the

authors in rewriting the manuscript. It has improved. For example, the hypotheses and

procedures are clearer now. I can say that I understand the study better now. However, the

manuscript still contains many major problems, and now that I understand the procedure and

analyses a bit better, new problems became evident. Most importantly, the regression models

fitted are not described in enough clarity (and at least one variable may be wrongly coded),

many claims are made based on wrong interpretations of 'interactions' and/or with no statistical

support (some examples below).

Additionally, I now see that what the authors call a 'windowed analysis' does not correspond to

what the term means by other authors using RQA because they did not use overlapping

windows. What the authors did was to divide the tapping time series into periods (which they

call windows 1, 2 and 3) and use these periods ('windows') as predictors in the regression

models to see if RQA measures wary across periods. I strongly suggest the authors do not use 'window' but find another term eg 'trial period' so as to distinguish from real windowed RQA.

For the common use of 'windowed' analysis, see for example:

Webber, C.L., Jr., Marwan, N. editors (2015).Recurrence Quantification Analysis: Theory and

Best Practices. Springer Series: Understanding Complex Systems. Springer International

Publishing, Cham, Switzerland. <http://dx.doi.org/10.1007/978-3-319-07155-8>).

- We agree with the reviewer’s point and have retitled “windowed MdRQA” to “region-based MdRQA.” We have updated the manuscript (and all associated files) accordingly.

The authors are strongly advised to discuss with a statistician how to interpret interactions in regression models.

- In our revision, we have attempted to provide more clarity for readers about how to understand our model specifications and results. Importantly, we have cited very useful work by Barr et al. (2013) that includes clear descriptions of deviation coding, conceptual discussions of its utility in linear mixed-effects models, and an appendix with detailed mathematical descriptions. We have further attempted to clarify the statistical underpinnings of our interpretations for our reader. (We note, too, that some effects did change once we recognized that our implementation of deviation coding for three-level factors was incorrectly accounting for the factors as though they were two-level factors, after the reviewer’s helpful review of our script.)

I will point to the major issues below. I feel I have devoted more than a reasonable amount of time helping improve this manuscript, so will not try to be exhaustive.

LINE 159

H2: The prediction is at odds with the fact that data from in-phase was excluded.

My understanding is that you do not need to compare successful vs unsuccesfull trial to test

the hypotheses. So, I don't follow the justification to exclude all in-phase data.

- Thanks for pointing this out. You are correct that trial type is not required for testing H2. As stated in the paper, analyzing trial type was a *post hoc* decision once we noticed the difficulty that many participants had with phasing. In order to create the most parsimonious model that accounted for important variability in the data (while simultaneously describing important but unanticipated differences between Successful versus Unsuccessful Trials), we included this variable so that our model could more faithfully capture real-world dynamics. We felt this was a more conservative approach to testing the hypothesis while also potentially accounting for important differences in trial dynamics, given that the model would still describe the variance uniquely accounted for by categorical relative phase.
- Unsuccessful Trials do not have enough in-phase data points to analyze synchronous regions using MdRQA. While Successful Trials technically have enough in-phase data points, Successful Trials make up only 38% of trials. Given this statistic and the fact that synchrony has already been extensively studied in previous literature, we chose to exclude synchronous taps so that we could include more than just 38% of our data.
- To clarify our intention for our readers, we have updated H2 as follows:

  “Participants will demonstrate more stable tapping near antiphase when compared to other nonsynchronous relative phases, due to the increased general stability of antiphase relations when tapping. *Operationalization:* This will be reflected by higher %REC, %DET, and MAXL during antiphase.”

LINE 259:

"350°"

I think you mean "359°"

- Yes, thank you for pointing out this error. We have corrected it in the most recent version.

LINES 269, 272, and others

You say 'categorical CRQA' but in this case the correct comparison would be 'continuous

CRQA.'

- Here, we were describing the relation of binary states of one variable (i.e., discrete metronome ticks; a tick or a not-tick at each time point) to the binary states of another variable (i.e., participant taps; a tap or a not-tap at each time point); CRQA between these two *particular* time series (tick, not-tick, tick, not-tick, etc.; tap, not-tap, not-tap, tap, not-tap, etc.) would then be categorical analysis. We attempted to clarify this with an added explanation to line 275.

  The reviewer is correct that categorical CRQA would be the correct way to describe the comparison between discrete metronome ticks and participant taps, which is what we are describing in lines 269 and 272. However, in the next paragraph, we attempted to describe why this would not be suitable for our data and why we therefore conducted MdRQA on continuous relative phase data. We have attempted to clarify this with an added explanation to line 275.

LINE 295:

"we were able to assess differences in recurrence, predictability, and attractor strength around

in-phase, antiphase, and between those regions"

You excluded the data around in-phase (in my view, unnecessarily), so you cannot assess

anything about it.

- As described in lines 296-305, Unsuccessful Trials did not have sufficient data points during in-phase synchrony to conduct MdRQA. Thus, we excluded in-phase data from all analyses to more effectively compare Unsuccessful Trials to Successful Trials.
- Additionally, because in-phase synchrony has been extensively studied and is a known global attractor, we chose to analyze nonsynchronous data because we wanted to focus on the nuances of phasing that occur between synchronous regions.
- We appreciate the reviewer’s suggestion to clarify our analyses. and have updated this line to say:

  “we were able to assess differences in recurrence, predictability, and attractor strength during nonsynchronous regions between the initial desynchronization and final resynchronization with the metronome. In other words, we focused on the system’s trajectory after it moved from synchrony, passed through antiphase, and approached synchrony again, rather than focusing on in-phase dynamics.”

LINES 316, Model specifications.

It is still unclear how the models were fitted.

Please distinguish the variables (success, language experience, tempo range, and 'window')

from the possible values (successful/unsuccesful, monolingual/multilingual, low/mid/high,

'window 1' /'window 2'/'window 3'), and indicate how the variables were coded before fitting the

model. For example, something like this: trial type was coded as a binary variable with

successful = 0 and unsuccessful = 1. Tempo range was coded as a categorical variable using

dummy coding…

- We have expanded the model specifications with a clarification of our coding scheme (i.e., deviation coding) and have added a footnote with specific values for each variable.

I looked at the R script and I do not understand what you mean by 'compliant' vs

'noncompliant.' Is this equivalent to successful/unsuccessful? Unclear.

- We apologize for this confusion. Initially, we referred to Successful and Unsuccessful Trials as Compliant and Noncompliant, respectively. We have updated the R script to reflect the current terminology.

I also believe there may be a serious mistake in how the tempo range data were coded.

Specifically, I believe mid-range tempo should probably be coded as lowerTempi == 0 and

upperTempi == 0 instead of lowerTempi == -.5 and upperTempi == -.5. Please check.

- We thank the reviewer for drawing attention to our coding scheme (here and in the earlier note). We are using deviation coding (rather than treatment or sum coding) in order to provide the most accurate estimation for linear mixed-effects models (cf. Barr et al., 2013). However, we incorrectly calculated it as a two-level factor (-1/2 and 1/2) instead of a three-level factor (-1/3, -1/3, and 2/3). We have corrected this error and updated the manuscript accordingly.

LINES 339, 348

"standard estimates"

I think you mean 'standardised' not 'standard'

- Thanks for noticing this error. We corrected “standard estimates” to “standardized estimates.”

Table 2

The table reports estimates for 'Successful trials' but if 'Successful trials' was the baseline what

is this estimate?

The table reports estimates for 'Language experience' but if monolingual language experience

was the baseline, this should be multilingual language experience, no?

The table reports estimates for an interaction between "Successful trials" and "Language

experience" but if successful and monolingual were the baseline, how do I interpret this

interaction effects?

Similar problems for the other interactions (e.g. Successful trials x Lower tempo)

Table 4 - similar issues to Table 2

- We have updated the tables and their captions to be clearer for our readers.
- As for interpreting the results, we recognize the difficulty in interpreting interaction terms among multiple coded variables. One of the most intuitive ways of interpreting these interactions is with plots, which is why we provide plots for all significant interaction terms. However, for those readers interested in understanding more about how to interpret the raw estimates, we now point them to excellent materials by Barr and colleagues (2013) that describe the mathematical and conceptual interpretation of deviation-coded variables. We hope this will provide readers with helpful guidance.

LINE 352

"This was partially supported by the general MdRQA results for %REC"

No. The results do not partially support H1.

- After updating the coding and analysis (as described above), we have re-interpreted and clarified the results (here and in the remainder of the paper). Our discussion now reflects that H1 is not supported.

LINE 353

"%REC was significantly greater during middle tempi than lower tempi"

No statistical evidence provided (eg posterior predictions) for this claim. Given the interactions,

you cannot simply rely on the 'fixed' (or 'main') effect of "Lower tempo range" to support this

claim. Fig 3 also does not provide evidence: the red/blue lines are incorrectly used, given that

tempo was binned and coded as categorical.

- Updating our analyses (as described above) to use deviation coding allows us to interpret lower-order main effects in the presence of higher-order interactions. Our updated general MdRQA results show that %REC and %DET do not significantly differ across tempi, while MAXL does.
- Thank you for mentioning tempo binning as it relates to Figure 3. We have updated all figures to clarify that tempo was binned for analysis purposes.

LINE 355

"Similarly, MAXL was significantly greater during middle tempi than lower tempi and during

upper tempi than middle tempi."

No evidence provided. Given the interactions, we cannot infer this from estimates reported in

Table 2 alone. And Fig 3C does not help either.

- Please see above response (to line 353).

LINE 372

"Trend lines indicate predicted results"

This is not the case, given that tempo was binned into three categories and these lines show a

continuous linear increase.

- In creating this figure, we were attempting to show a more holistic view of the data (in keeping with current data visualization best-practices to show all data). However, we agree that this could have led to a misinterpretation of our analyses by readers and have therefore updated the figure to reflect the binned tempo variable.

LINE 381

"...include the main effect of window on %REC..."

This is incorrect as it refers only to window 2, and does not consider the interactions.

- After updating the coding and analysis (as described above), we have re-interpreted and clarified this finding throughout the manuscript.

LINE 384

"The main effect of window on %REC revealed that window 2 had significantly lower %REC

than window 1, ..."

As above, given the interactions, this is not granted.

- We’ve clarified this in our results section, which we updated after properly implementing deviation coding.

LINE 387

"The interaction between trial type and window on %DET..."

This is only about window 3 not 2. This omission suggests the evidence is stronger than it

actually is. And this is in addition to being wrong way of interpreting interactions.

- We’ve re-interpreted this according to the implications of deviation coding.

LINE 387

"both Successful and Unsuccessful Trials yielded the highest %DET during window 2 and

smallest during window 3"

This is suggested by the summary stats in Table 3. But the link with the results from the

regression models is unclear, hence we do not know if these differences are 'significant.'

- In our updated results section, we’ve also re-interpreted this using language appropriate for deviation coding.

LINE 391ff

"The interaction between trial type and window on MAXL revealed 391 findings similar to those

for %DET. MAXL also peaked during window 2 for Unsuccessful Trials, which supports H2.

However, MAXL peaked during window 1 for Successful Trials, opposing H2. Both trial types

produced the smallest MAXL values during window 3."

Again, here the results from the regression ('interaction') is offered to support the claim that the

differences reported in the summary statistics are significant. But this is not granted.

This problematic interpretation of 'interactions' is repeated multiple times, e.g. L 413, L433,

(not exhaustive).

- As described in our responses above, we’ve updated our discussion of interaction terms to align with the implications of deviation coding.

LINE 436

"As measured by %REC, tapping data became less noisy as tempo increased"

This is not supported. This interpretation may be suggested by eyeballing Fig 3, but no

statistical evidence is provided, and even eyeballing Fig 3, the difference between monolingual

and multilingual seems minimal. Also, to say 'as tempo increased' is not consistent with the

fact that tempo was binned and treated as a categorical variable.

- This is supported by the significant interaction between language experience and tempo on %REC for general MdRQA. We hope that clarifying tempo binning in Figure 3, providing a detailed explanation of our variable coding scheme, and including resources on how to interpret main and interaction effects appropriately when using deviation coding helps the reader more clearly understand this finding.

LINE 436

"with multilinguals demonstrating a sharper and greater increase than monolinguals."

Not supported

- Please see our response to the previous line.

LINE 437

"These findings failed to support H3..."

Indeed the findings contradicted H3.
