# Supplementary material for: Multidimensional recurrence quantification analysis of human-metronome phasing
Source: PLoS One. 2023 Feb 23;18(2):e0279987. doi: 10.1371/journal.pone.0279987 (PMC9949643; doi:10.1371/journal.pone.0279987)
Supplement: Attachment — Submitted filename: Response to reviewers 12-18-22.docx [file pone.0279987.s006.docx]

Review Comments to the Author

Please use the space provided to explain your answers to the questions above. You may also include additional comments for the author, including concerns about dual publication, research ethics, or publication ethics. (Please upload your review as an attachment if it exceeds 20,000 characters)

Reviewer #2: Thank you for the opportunity to revisit this manuscript. The authors have put a lot of effort, and the analyses and their reporting are much improved and much clearer. I am happy to recommend that this manuscript be accepted for publication, and I think the few minor issues below can be addressed with the editor directly.

1. the sign for relative phase (psy) is not showing due to formatting to times new roman.

*The negative sign appears in the version we submitted. We followed PLOS One’s suggestion to use “Equation editor” in Microsoft word. Would it be possible for the editor to ensure the negative sign appears in the publication?*

2. the authors added citations to Barr et al 2013 but this work is not included in the reference list.

*Thanks for highlighting this mistake. We added Barr et al. (2013) as reference 51.*

3. there must have been a mistake along the way when producing the current version. In the response to the reviewer, the authors say they updated line 295 to include this new text:

“In other words, we focused on the systems trajectory after it moved from synchrony, passed through antiphase, and approached synchrony again, rather than focusing on in-phase dynamics.” But this passage does not appear in the amended manuscript.

*We edited the current revision to add this change. This line now appears at 297-299.*

4. About figures 3, 4, and 5. In the captions for all three figures, the authors say:

“trend lines indicate predicted results and confidence intervals of the linear mixed effects model, while individual points represent the raw data. Tempo was binned into lower, middle, and upper ranges for the analysis, while the raw data is presented at each individual tempo”

This would be perfect, but the trend lines are still incorrect in all cases. Given that the data was binned, the fitted models will produce predictions for three values of tempo: lower-range, middle-range, and upper-range. However the trend lines in the figures show predictions for 7 values corresponding to the 7 tempos in the raw data (80, 90, 100, 110, 120, 130, 140). It is not possible that the fitted models would have produced this linear increase (unless I am missing something). The authors should amend this figure to correctly represent the fitted models they report.

*To preserve the raw data while also making it clear that we binned tempi for analysis, we edited figures 3-5 by removing the trend line--which, as you pointed out, was not produced by our fitted models--and added violin plots of the binned tempo data. We updated figure 3-5 captions to state: “The metronome ranged from 80-140 bpm. In our linear mixed effects model, we binned tempo into the following categories: lower (80-90 bpm), middle (100-120 bpm), and upper (130-140 bpm). The violin plots depict the probability density of the binned tempo data at different %REC, %DET, and MAXL values. The mean value is indicated by a diamond near the center of each violin. Results of statistical analyses are located in Table 2.”*
